# Complete Genome Sequences of Three Uropathogenic Klebsiella quasipneumoniae Strains Isolated from Postmenopausal Women with Recurrent Urinary Tract Infection

**DOI:** 10.1128/MRA.00073-21

**Published:** 2021-03-18

**Authors:** Sundharamani Venkitapathi, Belle M. Sharon, Tahira A. Ratna, Amanda P. Arute, Philippe E. Zimmern, Nicole J. De Nisco

**Affiliations:** a Department of Biological Sciences, University of Texas at Dallas, Richardson, Texas, USA; b Department of Urology, University of Texas Southwestern Medical Center, Dallas, Texas, USA; Loyola University Chicago

## Abstract

Recurrent urinary tract infection (rUTI) poses a major health issue, especially among postmenopausal women. We report complete genome sequences of three Klebsiella quasipneumoniae strains isolated from the urine of postmenopausal women with rUTI. *K. quasipneumoniae* is a recently identified *Klebsiella* species with clinical and virulence characteristics distinct from those of K. pneumoniae.

## Abstract (precis)

Recurrent urinary tract infection (rUTI) poses a major health issue, especially among postmenopausal women. We report complete genome sequences of three Klebsiella quasipneumoniae strains isolated from the urine of postmenopausal women with rUTI. *K. quasipneumoniae* is a recently identified *Klebsiella* species with clinical and virulence characteristics distinct from K. pneumoniae.

## ANNOUNCEMENT

Recurrent urinary tract infection (rUTI), defined as two UTI episodes within 6 months or three within 12 months, poses a major health issue (1, 2). The genus *Klebsiella* is a leading cause of rUTI, accounting for 15% to 17% of cases (3). Klebsiella quasipneumoniae was originally associated with environmental niches; however, recent reports suggest a role in human infection (4). Lack of tests distinguishing *K. quasipneumoniae* from Klebsiella pneumoniae in clinical settings has led to a likely underestimation of *K. quasipneumoniae* prevalence (5).

Complete genome assemblies of uropathogenic *K. quasipneumoniae* isolates allow investigation of virulence and metabolic traits specific to this species. We report the complete genome sequences of three *K. quasipneumoniae* strains (Table 1) isolated from the urine of postmenopausal women meeting the criteria for uncomplicated rUTI as part of an institutional review board-approved study (STU032016-006, MR17-120) (6).

**TABLE 1:** Characteristics, assembly parameters, and accession numbers of three complete uropathogenic Klebsiella quasipneumoniae genome sequences

Strain	BioSample accession no.	SRA accession no.^*a*^	No. of raw reads	No. of trimmed reads	Read *N*_50_ (bp)	Read depth (×)	MLST^*b*^	GenBank accession no.	Type of contig (circular)	Total length (bp)	GC content (%)	No. of CDSs^*c*^	Plasmid replicon(s)
KqPF9	SAMN17016746	SRX9774966 (O)	150,239	148,741	11,189	153	UN^*d*^	CP065841	Chromosome	5,272,842	58.1	5,137	NA^*e*^
		SRX9779643 (I)	16,937,480	16,068,400		359		CP065842	Plasmid	399,394	48.3	435	IncFIB
								CP065843	Plasmid	4,730	42.6	6	Col(pHAD28)
								CP065844	Plasmid	4,096	55.5	5	Col440I
								CP065845	Plasmid	4,000	46.3	4	Col440I
KqPF26	SAMN17016750	SRX9774961 (O)	26,486	26,344	13,446	39	3387	CP065838	Chromosome	5,242,686	58	5,039	NA
		SRX9779644 (I)	12,077,320	11,492,042		271		CP065839	Plasmid	144,959	52.5	152	IncFIB(K), IncFII(K)
								CP065840	Plasmid	3,478	45.7	6	UN
KqPF42	SAMN17016751	SRX9774962 (O)	125,656	124,662	11,819	147	1535	CP065846	Chromosome	5,278,208	58	5,092	NA
		SRX9779645 (I)	18,822,828	17,989,506		420		CP065847	Plasmid	223,863	50.9	252	IncFIB(K)

a O, ONT; I, Illumina.

b MLST, multilocus sequence type.

c CDSs, coding sequences.

d UN, unknown.

e NA, not applicable.

Clean-catch midstream urine was obtained from three postmenopausal women, plated onto CHROMagar Orientation medium (BD), and incubated overnight at 37°C. Isolated single colonies were chosen for genus identification by PCR amplification and Sanger sequencing of the 16S rRNA gene, followed by a MegaBLAST query (BLAST v2.10.0) (6, 7) against the nonredundant/nucleotide (nr/nt) database. Species identification was performed by PCR using primers specific for the *K. quasipneumoniae* beta-lactamase (*bla*) and deoxyribose regulator (*deoR*) genes (8). *K. quasipneumoniae* genomic DNA (gDNA) was extracted from overnight cultures grown at 37°C in Luria broth (LB) using the gDNA extraction kit (BioBasic), followed by quality assessment using a 260/280-nm absorbance ratio and agarose gel electrophoresis. gDNA was sequenced using the Illumina NextSeq 500 system and the Oxford Nanopore Technologies (ONT) MinION platform. For Illumina sequencing, libraries were prepared using the Nextera DNA Flex library prep kit and sequenced to generate 2 × 150-bp paired-end reads. Illumina reads were quality assessed and trimmed using CLC Genomics Workbench v12.0.3 with cutoffs set at a minimum Phred score of 20 and a read length of 15 bp. Default parameters were used for all software unless otherwise specified. ONT libraries were prepared using a ligation sequencing kit (SQK-LSK109) and barcode expansion kit 13-24 (EXP-NBD114) and sequenced on R9 FLO-MIN106 flow cells. ONT MinKNOW v3.6.16 was used for base calling, demultiplexing, and barcode trimming. ONT read quality was assessed with NanoStats v1.2.0 (9), and reads were trimmed with NanoFilt v2.6.0 (9). Reads with a Phred score of >7 and a length of >200 bp were retained (Table 1).

The Illumina and ONT reads were used to construct hybrid assemblies of each strain, and the circular genomic sequences were rotated to the starting base of *dnaA* or *repA*, if present, using Unicycler v0.4.8 (SPAdes v3.13.0, Racon v1.4.10, and Pilon v1.23) (10–13). The quality of the hybrid assembly was evaluated using QUAST v5.0.2 (14), and the genome completeness was assessed with Bandage v0.8.1 (15) and BUSCO v1 (16) using the bacterial ortholog set on the gVolante server v1.2.1 (17). The NCBI Prokaryotic Genome Annotation Pipeline v4.11 was used for genome annotation (18, 19). The GC content and coding sequences were evaluated using Geneious Prime v2020.0.5. The sequence type was determined using MLST v2.0 (http://www.genomicepidemiology.org/) with the K. pneumoniae configuration (20). The plasmid replicons were identified with PlasmidFinder v2.1 (21, 22), using the *Enterobacteriaceae* database and default cutoffs (Table 1).

### Data availability.

The sequencing data were submitted to GenBank under BioProject accession number PRJNA683049. The BioSample and SRA accession numbers are reported in Table 1.
